# ‘Everyday genetics’ in the Mass Observation Project: insights on genetics from people writing for an archive of everyday life in Britain

**DOI:** 10.1038/s41431-026-02113-x

**Published:** 2026-04-29

**Authors:** Rachel Horton, Susie Weller, Lisa Ballard, Anneke Lucassen

**Affiliations:** 1 Centre for Human Genetics, University of Oxford, Oxford, UK; 2 Centre for Personalised Medicine, Centre for Human Genetics, University of Oxford, Oxford, UK; 3 Wessex Clinical Genetics Service, Princess Anne Hospital, Southampton, UK; 4 Primary Care, Population Sciences and Medical Education, University of Southampton. National Institute for Health and Care Research Biomedical Research Centre Southampton (Data, Health & Society Theme), Southampton, UK

**Keywords:** Ethics, Social sciences

## Abstract

Over the past two decades, genetic testing has undergone major shifts in its accessibility and in its nature. Historically, it primarily involved analysis of single genes selected on the basis of symptoms or family history, and was available only to a few. Now, options range from diagnostic clinical genomic tests, to broader screens offered to ‘healthy’ populations, to direct-to-consumer tests offering to explore ancestry. As genetic testing becomes an increasingly ‘everyday’ encounter, we sought to explore how the topics of genetics (and genomics) were considered in the Mass Observation Project, an archive of writing by ‘ordinary’ people about everyday life in Britain. 55% of the 147 respondents had personal experience of genetic testing or knew someone who had, typically to explore ancestry. Responses often gave the sense of genetic testing as a powerful tool in healthcare with results that were fairly definitive. Genomic testing was typically written about as an amplification of genetic testing, generating information of similar solidity. Writers threaded together personal experiences with insights drawn from a wide variety of media, often quite old, in outlining their ideas. While many positioned genetics as outside their remit, respondents engaged in depth with the opportunities and challenges raised, advocating for ethical/societal considerations to form a key part of decision-making regarding genetics. Our analysis shows that people without prior experience of clinical genetic testing may yet have a wealth of experiences and exposures sculpting their expectations as to what testing stands to bring. Consent conversations may benefit from exploring these.

## Introduction

As genetic and genomic tests become more widely available, many more people are engaging with the opportunities and challenges they raise. In the UK, the last fifteen years have seen the increasing use of genomic technology in rare disease diagnosis, from the Deciphering Developmental Disorders study [1], to the 100,000 Genomes Project [2], to the NHS Genomic Medicine Service [3]. In recent years, there has also been considerable interest around the role genomics could play in public health, with the Generation Study exploring this in the context of newborn screening [4], and Our Future Health considering the use of polygenic scores in assessing common disease risks [5]. Alongside this, direct-to-consumer genetic testing companies claiming to offer insights on aspects ranging from ancestry to wellness to disease risks have proliferated [6], and genomics has played an increasingly prominent role in political discourse [7–9].

In light of this, we aimed to explore how people with a rich experience of writing as part of the Mass Observation Project- ‘*an archive of everyday life, thought and feeling in Britain*’ [10]- appeared to conceptualise genetics. Mass Observation is a social research organisation founded in 1937. In 1981, it set up the Mass Observation Project, creating a panel of ‘Observers’ from across Britain, who are asked to write responses to prompts (‘Directives’) three times a year. These Directives are open-ended qualitative questionnaires, developed in conjunction with researchers, which cover a variety of topics. Some Observers contribute over many years, and over time may gain considerable experience of writing regarding topics outside their everyday experience, though ‘*ordinariness*’ and ‘*ordinary lives*’ remain central to the project [11]. As Haran and O’Riordan discuss, ‘*to write to the archive is to address the future and to carve out a place in the making of knowledge*’ [12].

The longitudinal nature of the project offered an opportunity to see how some Observers had engaged with genetics over time, as Haran and O’Riordan commissioned a directive about ‘*Genes, Genetics and Cloning*’ in 2006. They analysed responses over the following decade, reporting on the work in 2018 where they focused on ‘*how media cultures resource public knowledge-making over time*’, noting that responses indicated ‘*a pro-science and engaged public culture in relation to genetics in the UK*’ [12]. The scope and availability of genetic testing has since broadened, and such testing is also operating in a world changed by austerity, the pandemic, the internet and social media: we set out to explore how the Observers of 2022 wrote about genetics and genomics.

## Methods

### Development of the ‘Genetics and health in our everyday lives’ directive

We developed a directive- a questionnaire designed to generate rich, open-ended free text responses- entitled ‘*Genetics and health in our everyday lives*’ to gain a sense of what Observers thought about genetics and genomics. We designed a range of prompts encouraging Observers to draw on their own experiences and those of their friends and family to reflect on and write about genetics in our everyday lives and the issues genetic and genomic testing might raise. These questions were shared with the Mass Observation team and iteratively refined to create an engaging and accessible directive. In September 2022, the directive was sent to everyone on the project’s panel of Observers. Table 1 shows the aspects of our directive discussed within this paper (please see supplementary information for the full ‘*Genetics and health in our everyday lives*’ directive). Mass Observation directives do not have a closing date but this paper analyses responses submitted up until September 2023.

**Table 1:** Excerpts from the 2022 Mass Observation Project directive on ‘Genetics and health in our everyday lives’.

Genetic tests are being used more and more… from ancestry genetic tests that link long-lost relatives to plans to screen newborn babies with DNA tests. 20 years ago, genetic tests could only examine small portions of the genetic code, and this was technically difficult. Now tests can sequence a person’s entire genetic code (genome) quickly and cheaply. Sometimes this helps to diagnose a condition, or determine a treatment, but other times we don’t know what to do with the information found through such tests. Developments in genetics mean that medicine can sometimes be made more personalised to an individual, rather than a whole population.
•Please share what comes to mind when you think of ‘genetics’. You are welcome to write down words, phrases, or to include images or sketches. •Please tell us about **any experience you have had of genetic testing** including the experiences of those around you—family, friends, neighbours, colleagues, and others. You could include any test that has looked at your DNA (genetic code) either via the NHS or those bought direct from shops/online (e.g. Ancestry tests or 23andMe). •Are there any news reports, films, television programmes or books about genetics that have been of interest to you? If so, please share how or why. •What do you think might be the consequences (positive or negative) of being part of a DNA database? •How do you imagine genetics might feature in our lives in the future?
•We would be interested in hearing **your views** on [the UK Newborn Genomes Programme pilot study]. What might be the potential benefits and drawbacks? •Do you have any particular hopes or worries about the future uses of genetics?
(The full ‘*Genetics and health in our everyday lives*’ directive is available here: https://massobs.org.uk/wp-content/uploads/2024/06/125-Summer-2022-Grudging-Acts-Genetics-and-health-in-our-everyday-lives-The-Royal-Family-and-update-sent-12-09-2022.pdf).

### Data analysis

We thematically analysed the responses, seeking to identify key ways in which Observers conceptualised genetic and genomic testing, and sources that had been important in developing their ideas. Our analysis aimed to sit between the scientifically descriptive and artfully interpretive approaches outlined by Finlay [13].

Where developing a point suited to scientifically descriptive analysis, for example commenting on frequencies, we ensured that as regards this aspect we had coded responses in a systematic and reproducible way. Where the theme we were exploring involved more artfully interpretive analysis, for example looking beyond what Observers wrote to consider what ideas might be implicit in what they wrote, our emphasis was more on re-reading responses looking for examples and counter-examples to iterate and refine our ideas.

We read through Observers’ full responses, using NVivo (v15) to tag sections where people wrote of their ideas and experiences around genetics. Where responses collated by the Mass Observation project had been handwritten, we typed up relevant excerpts for ongoing analysis.

Next, we re-read all tagged material, developing codes to capture key ways in which Observers wrote about what they might expect from genetics, and noting any sources of information about genetics or genomics that they referenced. We developed some codes inductively based on what we noticed in the data, and some were influenced by Haran and O’Riordan’s 2018 report on the 2006 ‘*Genes, genetics and cloning*’ directive [12]. Through this process, we identified themes around how people were engaging with genetics and genomics and the issues they raise, and went back through the material to test and refine these themes further.

Each Observer uses a unique MO number, enabling responses between directives to be linked while preserving their anonymity. Where Observers responding to our directive had also responded to the 2006 directive we applied to access their 2006 response. In such cases, we read their 2006 response then their 2022 response immediately following, aiming to gain a sense of whether and how their ideas around genetics and genomics might have changed in the intervening years.

## Results

### Characteristics of observers responding to the ‘*Genetics and health in our everyday lives*’ directive

The ‘*Genetics and health in our everyday lives*’ directive was sent out to over 400 Observers of whom 147 responded, in keeping with the usual response rate to Mass Observation directives of 30–35%. The style of responses was very variable: some were typed, some handwritten; some were brief, others lengthy; some addressed the prompts in the directive point by point whereas others dealt with the topic more holistically. 22 Observers (15%) had previously responded to a 2006 Directive on ‘*Genes, Genetics and Cloning*’. 100 (68%) of the Observers were women and 94 (64%) were 60 or older, with many employed or previously employed in roles typically requiring higher education.

35 Observers (24%) responding to our 2022 Directive reported having had direct personal experience of genetic testing, with a further 45 (31%) reporting that a family member or friend had had genetic testing. In both cases, most tests had been to explore ancestry rather than for health reasons. In terms of medical tests, several Observers had had experience directly or indirectly with *BRCA* testing or some form of prenatal genetic testing. Two Observers wrote about a close family member having had genome sequencing to investigate health issues, and several others discussed experiences with tests that probably involved genome or exome sequencing. The proportion of responses indicating close proximity with genetic testing was high (in Harris et al.’s 2024 survey of genetic testing in the UK, 10% of people reported having had genetic testing [14]). This likely reflects that Observers for whom the directive topic felt personally relevant were more likely to respond, and that our directive encouraged Observers to write about the experiences of friends and family as well as their own.

Of the 67 Observers (45%) who did not describe having had a genetic test or knowing someone who had, several had evidently engaged in depth with decisions around whether to have testing, so lack of direct experience by no means equated to lack of engagement with relevant issues. For example, one Observer who had elected not to have testing wrote:

*We’ve been offered DNA testing as part of my son’s autism diagnosis … but with [named research] project haunting autistic spheres, this isn’t something I’m keen to embark on. I had been curious to investigate my own DNA from an ancestry perspective, but having read ‘How to Argue with a Racist’ by Adam Rutherford, which states that after a certain amount of time you’re not actually related to your own ancestors, I sort of wrote the whole thing off as an exercise in vanity. [M6759]*

In contrast, in the 2006 Mass Observation directive on ‘*Genes, genetics and cloning*’, just under 20% of respondents reported any kind of experience with genetic testing for inherited conditions, and ‘*the frequency of reports of direct experience of genetic testing for research purposes and paternity was very low*’ [12]. Out of the 22 Observers (15%) who responded both to our directive and to the 2006 Directive on ‘*Genes, Genetics and Cloning*’, 10 (45% of those who responded to both directives) wrote about new direct or indirect experiences with genetic testing in the intervening years: all were ancestry tests. Responses as a whole indicated increasing familiarity with genetic testing, and there were a couple of cases where an Observer had made a brief response in 2006 along the lines of ‘*doesn’t inspire me*’ or ‘*only a passing interest*’, and had gone on to write a lengthy and detailed response in 2022.

### The word ’genetics’ evoked a breadth of ideas

Our directive included the prompt ‘*Please share what comes to mind when you think of ‘genetics’.*While our directive focused on the use of genetics to inform healthcare, use in healthcare seemed to form a relatively small aspect of the responses by Observers to the word ‘genetics’. Some recounted school biology lessons and Mendel’s peas, and many described thinking of the double helix. Several people thought of laboratories and scientists:

*I think of long strings of codes and molecules, of lab technicians in white coats and microscopes. [A7000]*

*An image of a hospital laboratory, glass bottles, researchers in white. [W6667]*

*Years ago I saw a documentary about the discovery of DNA so I remember a scene in that where the scientists were marvelling at a 3D model of DNA. [W6757]*

However, perhaps more commonly, Observers framed genetics in terms of the passing on of familial traits, sometimes health-relevant but often not:

*…having my father’s green eyes, mother’s amazing bone structure and porcelain skin. [A2]*

*…having my Nana’s build, and my dad’s nose and my Grandma’s dimples. [G7708]*

Our Directive did not contain explicit references to forensic genetics, archaeological genetics, eugenics, designer babies or cloning, but these topics were clearly prominent in many Observers’ minds when thinking about genetics. Many people mentioned genetic testing as a ‘clincher’ in solving crime, sometimes in the context of TV dramas and sometimes in real life cases. However, a few Observers discussed that the presence of DNA might not equate to guilt, usually where they were describing potential concerns about participating in a DNA database.

Various Observers described ‘detective work’ with genetic testing in the context of tracing their family history. Several people referred to paternity tests on daytime TV, or programmes such as Long Lost Family [15]. Observers recognised genetic tests as being able to prove biological relationships, potentially uncovering family secrets, or confirming or disproving family histories drawn up based on historical records, although they were often scathing about attempts to ‘quantify’ ancestry.

Various Observers discussed the use of genetic testing in an archaeological context. A few Observers referenced the use of DNA testing in identifying the remains of Richard III, and in this case genetic testing was considered as providing indisputable evidence. As Nelkin and Lindee write in *The DNA Mystique*, ‘*[in stories built around exhumations], DNA resolves conclusively historical questions that were once dependent for their resolution on records and testimonies from those alive at the time … DNA is itself a historical text sometimes upstaging archaeological remains*’ [16].

Throughout many responses there was a sense that genetic testing was a powerful tool with results that were often definitive. As one Observer reflected: ‘*there’s something quasi-magical – alchemical might be a better word – about the instinctive popular attitude to DNA testing and the research into its practical uses. It’s hard not to think of it in very literal terms: that pulling a gene from one position to another (if such a thing is possible), or excising it, can immediately change physical appearance or personality traits, or directly influence the course of a disease or chronic medical condition. I’m sure that’s all fantasy, but it’s a creepy feeling nonetheless to feel oneself, in all one’s human complexity, reduced to…well, whatever a genome sequence looks like. I’m imagining it as a Manhattan Yellow Pages-thick tome filled with variations on ATGC*’ *[M3190]*.

### Genomic results tended to be written about as an amplification of genetic results

Some Observers reflected on changes in genetic testing over time. Often this involved personal experience of appropriate genetic testing becoming available, or recent testing making a previously elusive diagnosis for someone they knew. A shift from genetics to genomics was rarely formally discussed but many Observers reflected on the potential for broader testing to generate more information. Generally speaking, the sorts of results expected from genomic tests (such as the Newborn Genomes Programme [4]) were discussed in similar ways to the results of narrower tests, with an implicit expectation of similar clarity. For example, reflecting on the possibilities of newborn genome screening, Observers wrote:

*Whilst it could be devastating to discover, and at such a young age, that there could be the potential for a genetic disease, ultimately it can only be a good thing to learn this as soon as possible. We all hear constantly of how early diagnosis can be a life saver, and that would seem to fit in this context. [D6836]*

*I don’t think I’d want to know the death day of my newborn baby. [A1706]*

A few Observers explicitly referred to evolving abilities to interpret genomic variation, usually either in the context of close experience with medical genetic testing, or commenting on fluctuations they saw in results attempting to define their genetic ancestry. A small number of Observers directly discussed how their expectations of certainty from genetic testing had shifted over the years, and often in these cases described that a particular article or book had influenced this change:

*I have read articles which do claim on seeming good ground that some health issues have a genetic basis but now such articles tend to take a more cautious approach, not claiming that because someone has a particular variant of a gene that they will become ill, but that it increases the risk that they will. [M3231]*

Many Observers implicitly positioned knowledge around genomic variation as improving when writing about the benefits of participation in genomic databases. Although the mechanisms by which this would happen were not commonly spelt out, it was very widely recognised that a decision to contribute to a research database might help others in the future.

### Observers wondered how ‘genetic knowledge’ might change the human experience or the nature of humanity

In responses to a prompt asking about ‘*future uses of genetics*’, many Observers saw genetics as playing an increasing role in people’s lives:

*I think we can be certain that our quest to understand and fight disease will mean that our use of genetic testing and treatment will only become more and more normal for future generations. [F7368]*

Observers often associated genetic testing with increased longevity, but several wondered whether this would ultimately benefit humanity. Many Observers, when asked to comment on newborn genome screening, reflected on what it might be like to grow up with awareness of genetic predispositions from an early stage. These were often couched in quite fatalistic language but the concern that efforts to extend our longevity might simultaneously curtail our experience of living was very evident:

*Perhaps we should become more accepting as humans that something, probably passed through our genes, is going to kill us. I don’t necessarily want to know which particular disease is going to kill me. Maybe if we’re so focused on our own demise we forget to live life. [C3210]*

However, many Observers also discussed that early awareness of health risks might allow preventative treatment or result in better outcomes for some of the babies being screened. Various Observers also reflected that while it might be challenging where uncertainty is raised around a baby’s future health, ultimately people would adapt to and cope with this.

‘Knowledge’ of genetic predispositions was fairly often construed as a burden, and for many Observers, a key consideration was whether a person could take useful action in light of that knowledge. Some raised concerns about whether relevant treatments would in practice be accessible, and several were sceptical regarding the extent to which genetic findings would provoke meaningful lifestyle changes:

*Doing [genetic testing] for health reasons feels a little deterministic. I mean, maybe I’m predisposed to some sort of illness, but I’m going to die of something – my drinking predisposes me to liver failure, my smoking to lung and heart failure, my genes will be predisposing me to something else. What am I meant to do, pick one? ‘I’ll lay off the smoking because I fancy giving my genes a chance to kill me with testicular cancer instead’? The whole thing feels a little pointless. [J5734]*

The potential for increased use of genetics to be a pathway to eroding and distorting what humanity values was a common concern—as one Observer wrote, ‘*genetic information has the very real possibility to become not about the information but about how we can manipulate society and who and what we place worth, marginalisation, othering and importance on within humanity*’ *[F7710]*. 33 Observers (22%) made explicit reference to eugenics and/or the Nazis. These concerns were most often raised in the context of prenatal screening (which many people spontaneously discussed in response to prompts around newborn screening).

### Observers drew on material across many domains and over a long time period in detailing their opinions on genetics and its future uses

Reporting on the 2006 directive, Haran and O’Riordan noted that ‘*those epochs marked as different – the new genetics of the 1950s, the genome project of the 1980s and 1990s, and the genomics of the 21*^*st*^ *century – are not marked as distinct in respondents’ personal media cultures of genomics. People connect cultural production through the 20*^*th*^ *and 21*^*st*^ *centuries in ways that make sense to them*’ [12]. Similarly, in responding to our directive, Observers brought in experiences and exposures to genetics in different settings when discussing their perspectives around topics such as future uses of genetics or the merits of taking part in a DNA database. Concerns raised in ‘older’ stories were evidently still seen as pertinent. Table 2 lists media which observers referred to.

**Table 2:** Sources referred to in responses to ‘Genetics and health in our everyday lives’ directive.

Book	Film	TV	News story/ event
**1818: Frankenstein (Mary Shelley)** 1932: Brave New World (Aldous Huxley) 1947: The Diary of a Young Girl (Anne Frank) 1949: 1984 (George Orwell) **1968: The Double Helix (James Watson)** 1988: The House of Stairs (Barbara Vine) 2001: Seven Daughters of Eve (Bryan Sykes) 1987–2012: ‘Culture’ series (Iain M Banks) 1976–2021: Richard Dawkins’ books 2004: Life (Gwyneth Jones) 2005: Never Let Me Go (Kazuo Ishiguro) 2010: Immortal Life of Henrietta Lacks (Rebecca Skloot) 2014–2022: Adam Rutherford’s books 2016: The Industries of the Future (Alec Ross) 2019: The Age-Well Project (Susan Saunders) **2021: Klara and the Sun (Kazuo Ishiguro)** 2021: Ancestors (Alice Roberts)	1978: The Boys from Brazil **1993: Jurassic Park** **1997: Gattaca** 2005: The Island 2018: Three Identical Strangers	1993–1999: Star Trek: Deep Space Nine 1996-present: Silent Witness (genetic testing featured from 1998) 2004-present: Who Do You Think You Are? (genetic testing featured from 2006) 2009: The Incredible Human Journey 2010-present: Digging for Britain 2011: Botany: A Blooming History 2014: Tutankhamun: The Truth Uncovered 2015: Code of a Killer **2016: A World Without Down’s Syndrome?** **2011-present: Long Lost Family (genetic testing featured from 2019)** 2019-present: Forensics: The Real CSIs 2018–2020: A House Through Time 2021–2022: DNA Family Secrets	**1933–1945: Nazi Germany** **1996: Dolly the sheep** 2013: Angelina Jolie’s BRCA result **2013: discovery of remains of Richard III** 2018: report that Barbara Streisand has cloned dogs 2021: Genetic testing of Tarim mummies 2022: news reports about treatments for motor neuron disease

Items in bold were referenced at least three times.

Sometimes Observers made a reference but it was not clear which version of a story they were referring to (for example, whether they meant the book or the film for ‘Jurassic Park’): the classifications above reflect what we thought of first when seeing the reference.

None of the Observers referred by name to the 100,000 Genomes Project [2], except those who had some personal experience of it. While we asked Observers about the Newborn Genome Programme (now called the Generation Study) [4] in the directive prompt, many commented that they had not previously heard of it.

Although Observers referred to a wealth of material in their responses, this is not to suggest that they were blindly influenced by all materials in developing opinions regarding genetic testing in a healthcare context. Observers were clearly critically evaluating the sources of information they referred to and to what extent these might be sufficient to inform an opinion.

### Observers saw ethical expertise and societal involvement as essential in making decisions about genetics and its future uses

Although many more Observers have now engaged with genetic testing and the issues around it, many parallels remain with Haran and O’Riordan’s findings from 2006. In terms of attitudes towards their directive, they noted that ‘*some accounts included what could be characterised as an assertive lack of interest*’ [12]. The excerpt they used to illustrate this point started ‘*I am totally bored by this subject so that the amount I shall write will be very little [W3176]*’. In 2022, some Observers similarly resisted engaging with the directive. For example, one Observer’s complete response was:

*Why are we bothering to talk about genetics? Who will receive any benefit from it? The very rich who can afford expensive health care – maybe 5% of our population. [B4273]*

However, as above, where questions regarding the value of focusing on genetics were raised, this seemed more typically precipitated by concerns about inequities in benefit, rather than because an Observer felt the topic was boring. This aspect of disparate benefit is not prominent in Haran and O’Riordan’s report on responses to the (pre-austerity) 2006 Directive, but in 2022 the concern that genetics might be a vanity project that might distract from addressing basic issues appeared several times:

*Oh this is so expensive and luxurious and first world. The rich will have the test and worry and the poor won’t have the test and will worry more and the many parents in poor countries will continue to count themselves lucky if their babies survive malaria and malnutrition and the upcoming horrors of climate change. [S7592]*

Another factor common across several responses was people feeling ill-equipped to answer questions around genetics as they felt they did not have sufficient knowledge of the subject. For a few people, this meant they did not feel it worthwhile to engage with the directive. However, many Observers who did respond to various prompts still expressed reticence about their ability to give a helpful opinion because they perceived they had insufficient technical knowledge, with comments such as:

*Sorry, I am not really up on this subject, medical things go over my head … Feel free to bin my comments. [A2]*

In responses to the 2006 Directive, Haran and O’Riordan noted ‘*considerable evidence of deference to expert knowledge*’ but also found that ‘*even respondents positively disposed to scientists and research drew attention to risk and the need for regulation*’. While in responses to our Directive, ‘*deference to expert knowledge*’ was still often implied in that Observers would disclaim the value of their own opinion on the basis of insufficient expertise, it seemed that trust in technical scientific experts was very much contingent on demonstrable efforts to engage with ethical issues and listen to societal concerns:

*In general, genetic testing can provide insightful information but that information should be used wisely and be regulated by a panel of experts that has several experts in ethics. [W6724]*

*I think recent developments in genetic knowledge are remarkable. I think knowledge is still partial but the possibilities are wonderful. But it needs a strong ethical framework. [B6659]*

For the 22 Observers (15%) who had responded to both the 2006 and 2022 Directives, we were struck by how stable attitudes towards genetics appeared to be. Observers who had felt that they were undecided and easily swayed in 2006 felt the same in 2022; Observers who had been reticent continued to express reservations; those who had been broadly positive had retained their optimism about the future and potential of genetics. It is possible that Observers may have referred to and potentially been influenced by their previous responses in writing responses to our directive, though we did not make direct reference to the 2006 Directive in our prompts.

## Discussion

People writing for ‘*an archive of everyday life, thought and feeling in Britain*’ [10] on the topic of genetics, genomics and health drew together insights from a wide variety of sources in explicating their ideas and opinions. Over half of respondents had personal experience of genetic testing or knew someone who had, though often this had been to explore ancestry as opposed to for health reasons. Where asked to imagine genetics in a relatively undirected way, images that arose for many Observers occurred outside of healthcare but often in settings where genetics was expected to provide clear answers: inheritance patterns as shown by Mendel’s peas; analysis of DNA left at crime scenes; determining relatedness.

Genomics generally seemed to be written about as an amplification of genetics, with results of a similar nature expected, but on a grander scale. The potentially different nature of many genomic results received less prominence in the responses- for example, the subtlety of ‘high’ polygenic risk scores that represent a small increase in absolute risk [17, 18], or the uncertainty associated with hypothetically concerning rare variation identified via newborn screening of a ‘healthy’ baby [19, 20]. Where Observers discussed changes relating to genetics in healthcare over time, this was usually in a context of acknowledging improvements in test availability and interpretation which had led to new diagnoses or new options for them or people they knew.

Reflecting on future uses of genetics, many Observers recognised potential benefits in being made aware of genetic predispositions to disease, although many were also sceptical about the extent to which these might be realised, and concerned that the price could be too high in terms of the constraints such awareness might place on life. Implicit deference to expert opinion relating to genetics was common, with Observers frequently devaluing their own opinion because of perceived insufficient grasp of the topic, but this was caveated by a strong call for ethical expertise and societal considerations to be a key part of any decision-making regarding genetics and its future.

Our work links in with that of Roberts *et al*, who explored ‘*the resources that people have to make sense of science on their own terms*’ [21]. Drawing on De Certeau, they describe these resources as a person’s ‘*poaching territory- the cultural spaces to which they can go in order to take what they need to make sense of genetics*’. They caution how science communicators must beware of ‘*playing gamekeeper*’: where people draw on a particular cultural reference (they give the example of GATTACA), we should listen for what people are telling us through using it, rather than leaping to discuss the ways in which it is scientifically inaccurate [21]. Our directive asked people to describe and tap into their personal ‘poaching territories’ relating to genetics, and the epistolary nature of the Mass Observation Project allowed Observers to fully lean into this without the interference of a ‘gamekeeper’ curtailing their replies. The rich and nuanced responses we received illustrate the potential value of this approach. Interestingly, we also found that Observers seemed to be acting as their own gamekeepers – they drew on cultural resources (amongst other experiences) in particular contexts, for example, when envisaging a future where newborn genome screening was routine. However, they often explicitly evaluated these resources as likely to be technically inaccurate, and were drawing on them not for the science but for other purposes, for example, to caution around potential consequences of genomic testing that we must actively avoid.

### Strengths and limitations

The Mass Observation Project does not aim to be statistically representative of the UK population and we should not infer from it what ‘most’ people living in the UK might think about the topics of genetics and genomics, or the nature and extent of the sources and opportunities that might be available to them in developing their ideas. The degree of familiarity it indicates with genetic testing may partly reflect that Observers with relevant personal experiences were more likely to respond. Similarly, the wealth and timeframe of sources referred to may relate to the age demographic of Observers and the nature of their engagement with the Project across a wide range of different issues over time. However, as Haran and O’Riordan reflected in their report on the 2006 ‘*Genes, genetics and cloning*’ directive, the nature of the archive, with Observers discussing a very wide range of topics and contributing over many years, offers an opportunity ‘*to develop an understanding of what a small, socially engaged fraction of the UK population knew, believed and felt about contemporary developments in genomics, and perhaps of how they had come to form those knowledges, beliefs and feelings*’ [12]. The mode of data generation allows respondents to think about questions over time, and potentially research relevant issues or discuss them with others in the process of crafting a very considered response. Previous research indicates that the opportunity to absorb and reflect on more information can sometimes profoundly change people’s views in this area. For example, a public dialogue relating to the use of genetic testing in newborn screening for cystic fibrosis found that with information, discussion and time, a number of participants changed from favouring a sensitive approach (which prioritised detecting every baby with cystic fibrosis) to preferring a more specific approach (which would leave fewer families navigating uncertainty as to whether their baby would develop cystic fibrosis) [22].

## Conclusion

Our study indicates a trend towards increasing personal familiarity with genetics and genomics, but with older media, often created years before the advent of genomics, continuing to play into people’s ideas as to what genetics and its future might look like. It shows that people reading a participant information sheet, or attending for an appointment to discuss genomic testing, are unlikely to be attending with a ‘blank slate’ as regards their expectations from the test. This will also be the case for people buying direct-to-consumer genetic tests, or considering their perspective on population initiatives involving genomic screening. While provision of information might shift people’s views, they likely already have ideas regarding what results genetic testing might provide, potentially developed over many years and drawing on a very diverse range of experiences and sources. This highlights how important it is for people providing genomic tests to carefully explore and, where necessary, help calibrate expectations. Practices of seeking consent that focus purely on imparting information in the hope of informing decision-making may struggle to make much impact on rich ideas around the potential of genomic tests which may have been years in the making. By the same token, societal dialogues around roles for genomic screening in public health may flounder if they fail to acknowledge the thoughts people already have regarding its potential. Engaging with people to learn their personal starting point as to what they expect from genomic testing, and tailoring discussions accordingly, will help ensure robust consent and meaningful debate.

## Supplementary information

Full version of the 'Genetics and health in our everyday lives' directive

## Data Availability

Responses to the 2022 *Genetics and health in our everyday lives* and 2006 *Genes, genetics and cloning* directives can be viewed at The Keep, University of Sussex, or online by application to the Mass Observation Project team (https://massobs.org.uk/).
