# Supplementary material for: ‘Everyday genetics’ in the Mass Observation Project: insights on genetics from people writing for an archive of everyday life in Britain
Source: Eur J Hum Genet. 2026 Apr 29;34(6):803–9. doi: 10.1038/s41431-026-02113-x (PMC13247082; doi:10.1038/s41431-026-02113-x)
Supplement: Supplementary file 1 — Full version of the 'Genetics and health in our everyday lives' directive [file 41431_2026_2113_MOESM1_ESM.docx]

Genetics and health in our everyday lives

**For this Directive we are interested in your views and experiences of genetics and health.**

Genetic tests are being used more and more … from ancestry genetic tests that link long-lost relatives to plans to screen new-born babies with DNA tests.

20 years ago, genetic tests could only examine small portions of the genetic code, and this was technically difficult. Now tests can sequence a person’s entire genetic code (genome) quickly and cheaply. Sometimes this helps to diagnose a condition, or determine a treatment, but other times we don’t know what to do with the information found through such tests.

Developments in genetics mean that medicine can sometimes be made more personalised to an individual, rather than a whole population.

# Your experiences of healthcare

Thinking about your own experiences of healthcare (this does not need to include genetic testing):

- Can you describe a time when you felt you **experienced care that was personalised** or tailored to your own specific situation?
- We would also like to hear about experiences of care that **did not** feel personalised to your own circumstances.
- Have you ever felt that any aspects of your identity or background have affected your experience of healthcare?
- On what basis do you think medical care should (or should not) be tailored to you? Please share your feelings on this.

# Genetics in our everyday lives

Please share what comes to mind when you think of ‘genetics’. You are welcome to write down words, phrases, or to include images or sketches.

Please tell us about **any experience you have had of genetic testing** including the experiences of those around you – family, friends, neighbours, colleagues, and others. You could include any test that has looked at your DNA (genetic code) either via the NHS or those bought direct from shops/online (e.g. Ancestry tests or 23andMe).

Are there any news reports, films, television programmes or books about genetics that have been of interest to you? If so, please share how or why.

# The issues genetic testing can raise

We would like to hear your thoughts on the following fictional scenario:

*M has genetic testing after developing breast cancer. Her test finds she had a strong chance of developing breast cancer. M’s doctor says that her sister could be tested for this inherited tendency (and if she has it, she could have extra screening or surgery to reduce her risks). M has not told her sister about her breast cancer and does not want to tell her sister about the genetic result. Her sister has a 50:50 (1 in 2) chance of having a strong genetic tendency to breast cancer.*

- What do you think about this?
- Do you think M’s sister should be told? If so, who should tell her?
- Should M’s doctor communicate the potential risks to her sister if she doesn’t?
- M has agreed for her genetic data to be stored in a DNA database and used for research purposes. What do you think might be the consequences (positive or negative) of being part of a DNA database?

# Future uses of genetics

How do you imagine genetics might feature in our lives in the future?

One potential future use is the UK **Newborn Genomes Programme** pilot study, which will examine the benefits and drawbacks of sequencing a baby’s entire genetic code then analysing parts of it to look for various genetic conditions. It could detect more conditions than the current newborn ‘heel prick’ test. However, the programme might also cause more uncertainty (for example, where a possible genetic risk is found but no-one is sure if it will make the baby ill or it is unclear what the best treatment option is).

We would be interested in hearing **your views** on this initiative. What might be the potential benefits and drawbacks?

Do you have any particular hopes or worries about the future uses of genetics?
